# Construction of a risk model associated with tryptophan metabolism and identification of related molecular subtypes in laryngeal squamous cell carcinoma

**DOI:** 10.3389/fgene.2025.1530334

**Published:** 2025-03-24

**Authors:** Feng Liu, Yanchao Qin, Wei Luo, XianHui Ruan, Lifang Lu, Bowei Feng, Jianfei Yu

**Affiliations:** ^1^ Department of Head and Neck Surgery, Shanxi Cancer Hospital, Taiyuan, China; ^2^ Department of Thyroid and Neck Tumor, Tianjin Medical University Cancer Institute and Hospital, National Clinical Research Center for Cancer, Key Laboratory of Cancer Prevention and Therapy, Tianjin’s Clinical Research Center for Cancer, Tianjin, China; ^3^ School of Stomatology,Shanxi Medical University, Taiyuan, China; ^4^ Department of Head and neck radiotherapy, Shanxi Cancer Hospital, Taiyuan, China

**Keywords:** tryptophan metabolism, laryngeal squamous cell carcinoma, bioinformatic, risk model, immune

## Abstract

Catabolic metabolites of tryptophan (Trp) are considered to be important microenvironmental factors by suppressing anti-tumor immune responses in cancers. Nevertheless, the effect of Trp metabolism (Trp metabolism)-related genes Trp metabolism-related genes on laryngeal squamous cell carcinoma (LSCC) progression is not yet clear. So, in this study, the TCGA-LSCC, GSE27020, and 40 TMRGs were extracted *via* public databases to explore the effects of TMRGs on laryngeal squamous cell carcinoma. Firstly, Weighted Gene Co-expression Network Analysis (WGCNA) was adopted with LSCC samples in TCGA-LSCC to acquire key module, and differentially expressed genes between LSCC and normal samples from TCGA-LSCC were yielded *via* differential expression analysis. Next, differentially expressed TMRGs (DE-TMRGs) was obtained in key model and DEGs, and prognostic genes were identifde through multiple algorithms. Five prognostic genes, namely *SERPINA1*, *TMC8*, *RENBP*, *SDS* and *FAM107A* were finally identified. A risk model was established based on the expressions of prognostic genes and survival information of LSCC samples while that were divided into high and low risk groups. Obviously, the LSCC immune dysfunction and exclusion score of high-risk patients was dramatically higher than that in low-risk patients, indicating that patients in the high-risk subgroup exhibited reduced responsiveness to immunotherapy. Besides, the drug sensitivity analysis showed that the low -risk subgroup was notably sensitive to Salubrinal, Lenalidomide, Metformin, while high -risk subgroup was more responsive to Docetaxel, AUY922, Embelin. Eventually, two clusters of LSCC samples had notable correlations with LSCC prognosis. The above results indicated that the risk model consisted of TMRGs (*SERPINA1*, *TMC8*, *RENBP*, *SDS* and *FAM107A*) was constructed in LSCC, contributing to studies related to the prognosis and treatment of LSCC.

## 1 Introduction

Laryngeal cancer (LCa) is the second most prevalent malignant tumor affecting the head and neck region, with an incidence rate second only to oral carcinoma, accounting for about 1.98% of all malignancies in the whole body, and in 2020 there was about 180,000 new patients with LCa and nearly 100,000 deaths worldwide (Sung et al., 2021; Atef et al., 2019). Squamous cell carcinoma (SCC) accounting for about 90% of LCa, is the predominant LCa subtype (Megwalu and Sikora, 2014; Siegel et al., 2020). The pathogenesis of laryngeal squamous cell carcinoma (LSCC) is still unclear, and related studies have shown that smoking, drinking, environmental factors, gastroesophageal reflux, HPV infection, radiation and other factors are associated with LSCC, which may be the result of a multifactorial interaction (Steuer et al., 2017). With the development of endoscopic and imaging technologies, the diagnosis and treatment of LSCC have been improved significantly. However, due to the lack of typical symptoms in the initial phase, approximately 60% of patients diagnosed with LSCC are already in advanced stages of tumor development. This delay in diagnosis hinders timely treatment and subsequently leads to unfavorable clinical outcomes (Strojan et al., 2013; Calhoun, 1988; Gao et al., 2019). In addition, despite the continuous improvement in surgery, chemotherapy and radiotherapy, the recurrence and metastasis rates of LSCC are still high and impose a serious impact on prognosis (Haddad et al., 2018; Gao et al., 2020; Bray et al., 2018; Lu et al., 2018; Nocini et al., 2020). In recent years, it has been pointed out that a possible way to improve the diagnostic and therapeutic effects of LSCC is the use of effective biomarkers, especially amino acid metabolic-related markers, which play an increasingly important role in the early detection of malignancies and the identification of potential immunotherapeutic targets (Zhang et al., 2018; Liu et al., 2023), but have not yet been well explored in LSCC. Therefore, the identification of effective biomarkers is important for the prediction of prognosis and guidance of personalized therapy in LSCC patients.

Tryptophan (Trp) is a crucial amino acid, constituting approximately 1% of the overall amino acids. It is not biosynthesized within our body and can solely be acquired through dietary intake (Wang et al., 2015). In the body, Trp is metabolized mainly through the Kynurenine pathway (KP) to produce downstream metabolites such as Kynurenine (KYN). Indoleamine 2,3-dioxygenase 1 (IDO1), indoleamine 2,3-dioxygenase 2 (IDO2), and tryptophan 2,3-dioxygenase 2 (TDO2) are the key rate-limiting enzymes for Trp metabolism. Numerous research studies have consistently demonstrated the significant upregulation of IDO1 in diverse tumor types, such as melanoma, cervical cancer, oral squamous cell carcinoma, colorectal cancer, esophageal cancer, and pancreatic cancer (Zhai et al., 2018; Chen et al., 2020; Prendergast et al., 2014). Furthermore, the presence of IDO1 expression in either tumor cells or stromal cells has been linked to an enhanced aggressive phenotype, unfavorable clinical outcomes, and a bleak prognosis (Zhang et al., 2022; Iversen et al., 2015; Rubel et al., 2018; Kozuma et al., 2018; Hornyak et al., 2018). Previous research has indicated that the metabolism of Trp in cancerous cells and/or surrounding cells plays a role in inhibiting the immune system’s ability to fight against tumors (Platten et al., 2021). Trp and its metabolites interact with IDO can modulate not only tumor cells, but also the entire tumor microenvironment, and ultimately promoting immunosuppression and drug resistance (Greene et al., 2019; Li and Zhao, 2021; Maleki et al., 2015). In addition, Trp metabolism, a novel immune checkpoint that can influence the outcome of immunotherapy, is also a potential therapeutic target (Li et al., 2019). From the above mentioned studies, it is known that Trp metabolism plays a pivotal role in initiation and progression of various cancers; however, the role of Trp metabolism-related genes (TMRGs) in LSCC and their prognostic value are not yet clear.

In this study, we extracted TCGA-LSCC, GSE27020, and 40 TMRGs from publicly available databases, and then identified DE-TMRGs by Weighted Gene Co-expression Network Analysis (WGCNA), differential expression analysis, univariate Cox, least absolute shrinkage and selection operator (LASSO), and multivariate Cox regression analysis, and finally combined with relevant clinicopathological information to construct a risk model of Trp metabolism. In addition, we further analyzed the impact of the risk model on the immunotherapy and chemotherapy for LSCC. We also identified the optimal clustering form (molecular subtypes) by consensus clustering analysis of samples from TCGA-LSCC, and explored the relationship between clustering and LSCC prognosis. Therefore, this study aims to construct a risk model by analyzing the TMRGs in LSCC to further predict the prognosis of LSCC patients and guide personalized treatment.

## 2 Materials and methods

### 2.1 Sources of data

The RNA-seq data, copy number, and somatic mutation data for TCGA-laryngeal squamous cell carcinoma (LSCC) were obtained *via* University of California Santa Cruz (UCSC) Xena (http://xena.ucsc.edu/), and a total of 111 LSCC samples and 12 control samples (82 LSCC samples with disease-free survival (DFS) information and clinical information) from Larynx were selected as the TCGA-LSCC dataset. The GSE27020 dataset consisting of 109 LSCC samples with DFS information was gained from Gene Expression Omnibus (GEO) (https://www.ncbi.nlm.nih.gov/), and was sequenced by GPL96 paltform. The GSE143224 dataset (GPL5175) consisting of 11 control samples and 14 LSCC samples was also gained from GEO. Then, a total of 40 TMRGs were extracted *via* Molecular Signatures Database (MSigDB) (https://www.gsea-msigdb.org/gsea/msigdb/index.jsp) (Xue et al., 2022).

### 2.2 Copy number and somatic mutation analyses

In TCGA-LSCC, the wilcox. test was utilized to analyze the differences of TMRGs expression between LSCC and normal samples. After that, the somatic mutation frequency and copy number variant (CNV) frequency of TMRGs in LSCC samples were analyzed by R package maftools (v 2.2.10) (Xu Q. et al., 2021), and the location information of TMRGs in chromosomes was explored *via* R package RCircos (v 1.2.2) (Zhang et al., 2013). To further understand the correlations among TMRGs, the expressions of TMRGs in LSCC samples were extracted, and Spearman correlation analysis was carried out.

### 2.3 WGCNA and differential expression analysis

For the purpose of obtaining the key module that related to Trp metabolism (TM), the WGCNA was applied in TCGA-LSCC. First, the TM score of LSCC samples in TCGA-LSCC was scored utilizing the Gene Set Variation Analysis (GSVA) algorithm according to TMRGs, and which was considered as trait for WGCNA. Outliers were removed by clustering the samples to ensure the accuracy of the analysis. A soft threshold was determined to optimize the conformity of gene interactions with a scale-free distribution. Subsequently, the dissimilarity coefficient was introduced based on the adjacency and similarity between genes, leading to the generation of a systematic clustering tree for genes, and modules were screened out according to the dynamic tree cutting criteria. What’s more, the module with the highest correlation with TM score was sifted out as the key module. Finally, differentially expressed genes (DEGs) between LSCC and control samples from TCGA-LSCC were screened out by R package edgeR (v 3.36.0) (Liu S. et al., 2021) by setting |log_2_FC| > 1 and adj. *P* < 0.05, and they were overlapped with genes in key module to yield differentially expressed TMRGs (DE-TMRGs).

### 2.4 Construction of a risk model

In order to identify genes that exhibited significant associations with survival outcomes in TCGA-LSCC, a univariate Cox analysis was conducted on DE-TMRGs, focusing on genes with a *P* value less than 0.05. Then, the LASSO analysis of genes gained in above step was applied by R package glmnet (v 4.0-2) (Zhang et al., 2019) to yield genes corresponding to lambda min, and they were analyzed for multivariate Cox to identify prognostic gene. A risk model was constructed according to the expressions ( expr ) of prognostic gene and risk coefficient ( coef ) in multivariate Cox. After calculating the risk score for the LSCC samples in the TCGA-LSCC ( $\text{Riskscore}=\sum_{i=1}^{n}\text{coef}\left({\text{gene}}_{i}\right)*\text{expr}\left({\text{gene}}_{i}\right)$ ), the samples were separated into high and low risk subgroups in accordance with their median value. Kaplan-Meier (K-M) survival analysis was used to compare the variations in survival between these two subgroups. Thereafter, we created a receiver operating characteristic (ROC) curve using survivalROC package (v1.0.3) in order to evaluate the risk model’s predictive accuracy (Zheng et al., 2021). Meanwhile, the difference of risk score between Disease Free and Recurred/Progressed groups was analyzed by wilcox. test, and the chi-square test was applied to determine the percentage of Disease Free and Recurred/Progressed patients in the high/low-risk subgroups. Furthermore, the findings were validated by the identical methods in GSE27020.

### 2.5 Nomogram construction and functional enrichment analysis

To further investigate the relationship between risk score and clinical features of 82 LSCC samples in TCGA-LSCC (age, overall survival (OS), sex, T, N, grade, stage, and DFS), a violin diagram was drawn, and the expression of prognostic genes in various risk subgroups and clinical features was investigated using a heat map. In TCGA-LSCC, risk score and clinical features (age, grade, M, T, N, sex, and stage) were analyzed by univariate Cox analysis, and factor meeting *P* < 0.05 was defined as the independent prognosis factor. After that, a nomogram was constructed by rms package (v 6.2-0) (Liu TT. et al., 2021) to forecast the likelihood of patient survival. Additionally, to further validate the nomogram, ROC curves, calibration curves, and decision curve analysis (DCA) were run. Ultimately, the risk model was also subjected to hallmark pathway enrichment analysis to further explore the biological functions and pathways (Background gene set: Hallmark: h. all.v7.4. symbols. gmt).

### 2.6 Immune infiltration analysis and drug sensitivity analysis

In the high/low-risk subgroups from TCGA-LSCC, we adopted the single-sample gene set enrichment analysis (ssGSEA) algorithm to estimate the levels of immune cell infiltration, and intersubgroup differences of them were compared *via* wilcox. test. The Spearman correlation between risk score and immune cells was also examined, and variations in conventional immune checkpoints of subgroups were compared. What’s more, the LSCC tumor immune dysfunction and exclusion (TIDE) value was calculated, and difference of it between subgroups was contrasted. To investigate the impact of risk model on chemotherapy for LSCC, an analysis was conducted to assess 138 chemotherapy drugs sensitivity of LSCC in cancer (GDSC) database (https://www.cancerrxgene.org/), and the differences in 50% inhibitory concentration (IC_50_) of 138 chemotherapy drugs were estimated between subgroups.

### 2.7 Consensus clustering analysis

For further assessing the role of prognostic gene in clinical classification of LSCC, clustering of LSCC samples from TCGA-LSCC was performed. First, using the ConsensusClusterPlus package (v 1.62.0) (Dong et al., 2021), consensus clustering analysis was carried out on 82 LSCC samples with survival data based on the prognostic gene., and the optimal clustering form was obtained based on the cumulative distribution function (CDF) value. The K-M analysis was adopted to study the association between clusters and LSCC prognosis. Subsequently, the differences of above immune cells, conventional immune checkpoints, TIDE value, as well as IC_50_ of 138 chemotherapy drugs between clusters were also analyzed.

### 2.8 Verification of expression level and RNA isolation and quantitative real-time polymerase chain reaction (qRT-PCR)

In GSE143224, Wilcoxon test was performed to analyze the prognostic genes expression in tumor and normal groups (p < 0.05).

Subsequently, to further analyze the prognostic genes expression in clinical samples, we conducted qRT-PCR. A total of 10 pairs of normal and LSCC samples were obtained from the clinic in the Shanxi Provincial Cancer Hospital. All participants were given informed consent. The study had the approval of the Shanxi Provincial Cancer Hospital ethics committee.

Total RNA of 20 samples were extracted by employing the TRIzol reagent (Ambion, United States) as per the manufacturer’s instructions. The SureScript First-strand cDNA Synthesis kit (Servicebio, China) was utilized to perform reverse transcription and generate cDNA. For qRT-PCR analysis, a CFX Connect Thermal Cycler (Bio-Rad, United States) was employed. The 2^−ΔΔCT^ method was utilized to ascertain the relative quantification of mRNAs (Livak and Schmittgen, 2001). The sequence information for all primers was displayed in Table 1.

**TABLE 1:** The sequence information for primers.

Gene name	Primer sequence (5′-3′)
SDS F	CTG​CCC​AAG​ATC​ACC​AGT​GT
SDS R	TTC​GGA​GAT​TCC​CCT​CCA​GT
FAM107A R	CTG​AGG​CAG​CTG​GGA​AAT​GA
SERPINA1 F	AAT​TCC​AGG​TTG​GAG​GGG​CG
SERPINA1 R	ACG​GCA​TTG​TCC​TGT​GGA​AC
TMC8 F	CCT​GAA​CTT​GAC​CCT​CCA​GTG
TMC8 R	CCC​TGC​CAG​TCA​AAA​CAT​GC
RENBP F	TGG​CTT​CCT​AGA​GGA​GCA​GGA
RENBP R	GCT​TCA​GAC​GTC​ACC​ATC​CC
Internal references -GAPDH F	CGA​AGG​TGG​AGT​CAA​CGG​ATT​T
Internal references -GAPDH R	ATG​GGT​GGA​ATC​ATA​TTG​GAA​C

### 2.9 Statistical analysis

The R software was utilized for conducting statistical analysis. The Wilcox test was employed to examine the variations among the groups. When P < 0.05, a substantial difference was deemed to exist.

## 3 Results

### 3.1 The genetic landscape of TMRGs in LSCC samples

**FIGURE 1:**
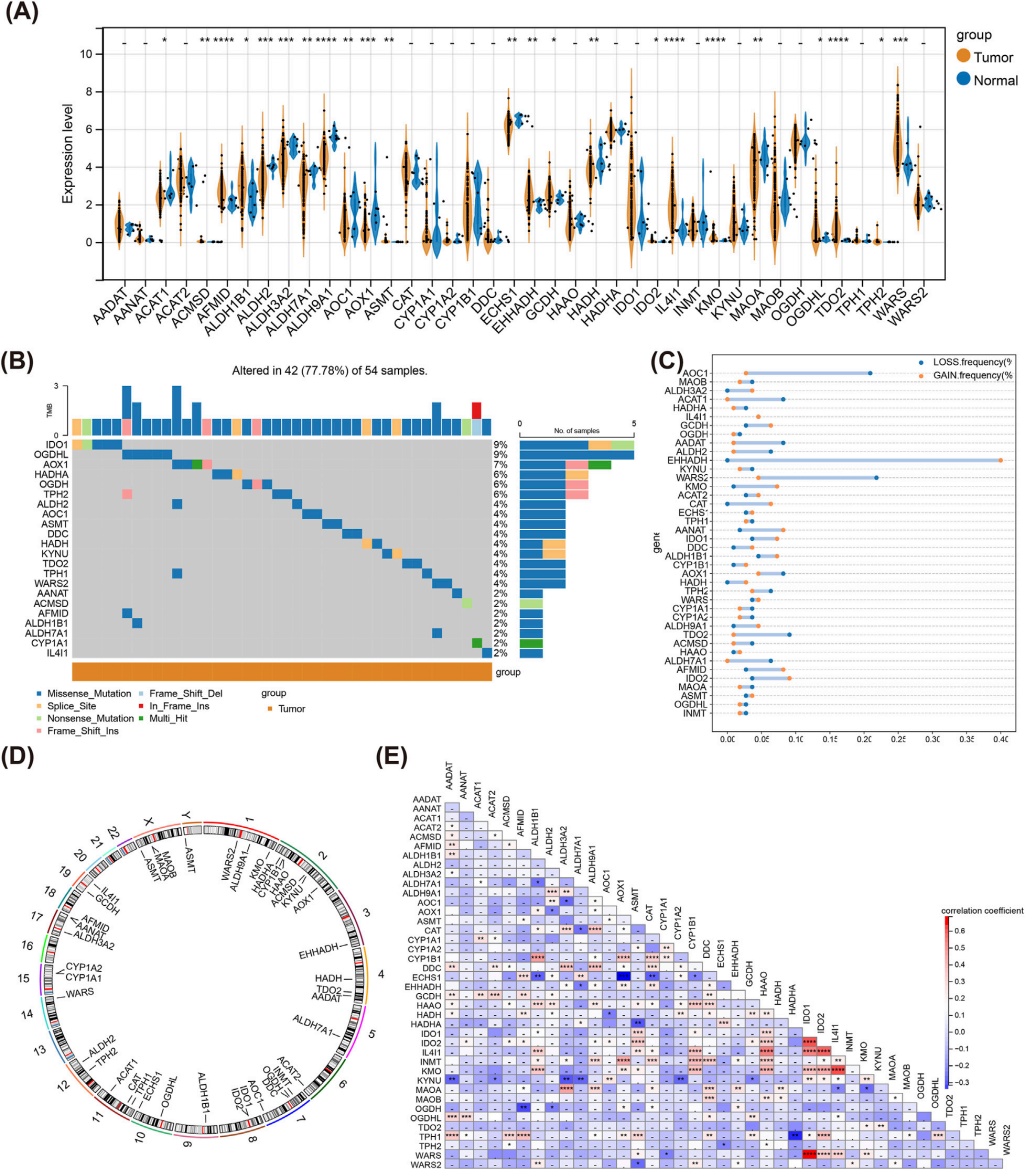
Genetic landscape of TMRGs in LSCC. **(A)** Differential expression of TMRGs between tumor and normal tissue samples. **(B)** Somatic mutational map of TMRGs in tumor samples. **(C)** CNV of TMRGs within tumor samples. **(D)** Infosphere diagram of TMRGs at different chromosomal locations. (where the gene ASMT occurs on both chromosomes X and Y). **(E)** Heatmap of correlation between TMRGs. * means p < 0.05,** means p < 0.01,*** means p < 0.001,**** means p < 0.0001, - means p > 0.05. TMRGs, tryptophan metabolism-related genes; LSCC, laryngeal squamous cell carcinoma; CNV, copy number variant.

Violin plot revealed that totally 23 TMRGs were notably differentially expressed between LSCC and normal samples (Figure 1A). Besides, IDO1 and OGDHL had high mutation frequencies in LSCC somatic mutations, and the main mutation type was Missense Mutation (Figure 1B). In LSCC samples, EHHADH exhibited the highest frequency of copy number amplification and WARS2 the highest frequency of copy number deletion (Figure 1C). Figure 1D illustrated the location of TMRGs on different chromosomes, where ASMT located on both chromosomes X and Y. Eventually, there was a notable strongest positive correlation between IDO1 and WARS, and AOX1 had a significant strongest negative correlation with ECHS1 (Figure 1E).

### 3.2 Identification of key module and DEGs

**FIGURE 2:**
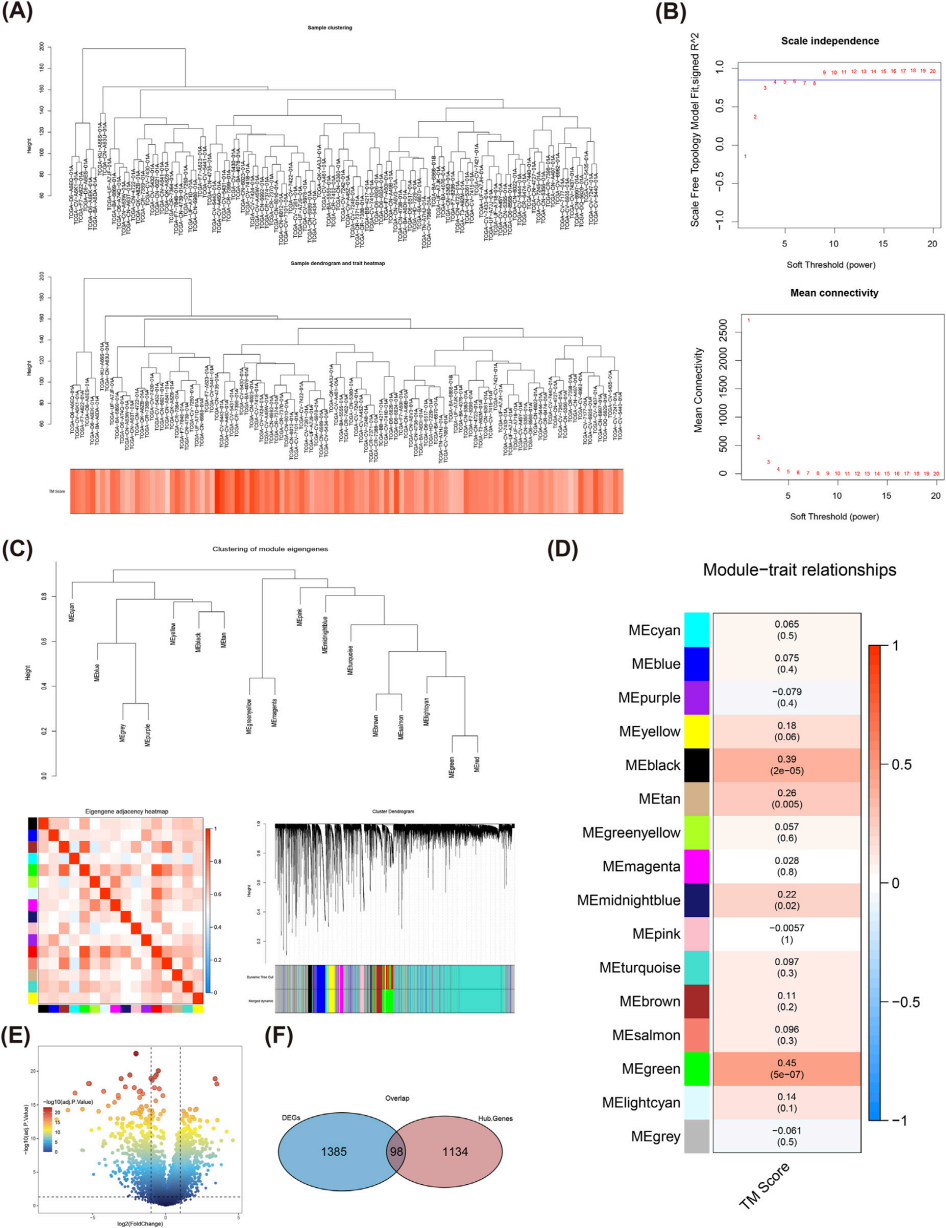
WGCNA analysis of TMRGs in TCGA-LSCC samples. **(A)** The overall clustering of TCGA-LSCC samples. **(B)** Scale-free soft threshold distribution and determines the power threshold value to be 9. **(C)** The Clustering of module eigengenes; Eigengene adjacency heatmap; Module clustering dendrogram. **(D)** Heat map of module correlation with clinical characteristics, the green module has been selected as a key module, containing 1,232 module genes. **(E)** Gene expression difference between LSCC and normal samples is displayed by a volcano plot. **(F)** A total of 98 DE-TMRGs were determined among the differentially expressed genes and module genes. TMRGs, tryptophan metabolism-related genes; LSCC, laryngeal squamous cell carcinoma; CNV, copy number variant.

The clustering of TCGA-LSCC samples showed that there was no necessity to exclude any samples (Figure 2A). Based on Figure 2B, a soft threshold was chosen as 9. Meanwhile, the network was approaching scale-free distribution, as seen by the vertical coordinate R^^2^ roughly converges around 0.85 and the average value of the adjacency function steadily goes towards 0. After that, the 16 modules were finally sifted out according to co-expression matrix (the similar modules had been merged) (Figure 2C), among which the green module was defined as the key module, which exhibited the highest correlation with TM score (Cor = 0.45, and P < 0.05), and which contained 1,232 module genes (Figure 2D). There were 1,483 DEGs between LSCC and normal samples (Figure 2E), and they were intersected with genes in key module to get 98 DE-TMRGs (Figure 2F).

### 3.3 The risk model showed an excellent ability for predicting prognosis of LSCC patients

**FIGURE 3:**
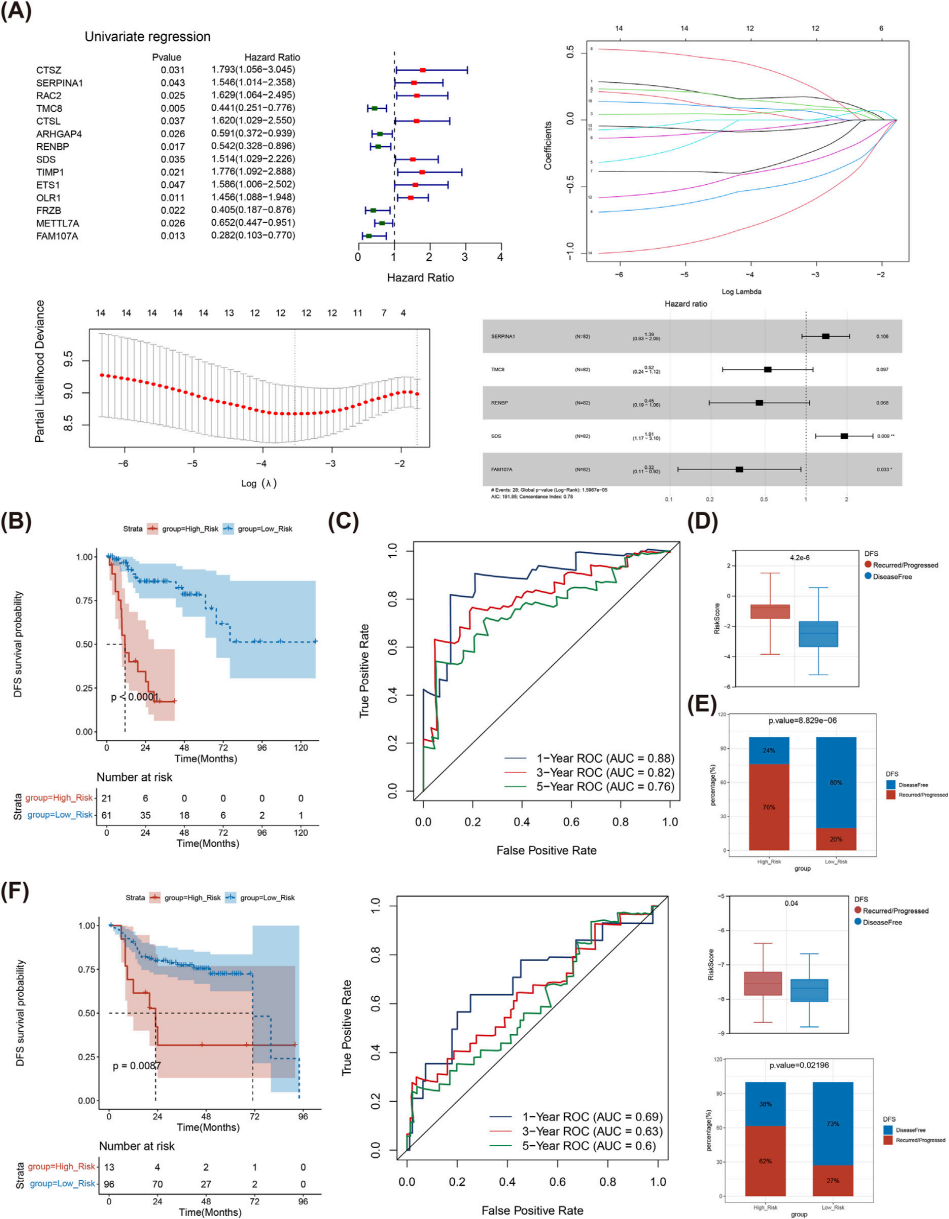
Determination of genes contributing to risk model. **(A)** Univariate Cox analysis of DE-TMRGs; Trajectory schemes were drawn for every independent variable associated with lambda; Confidence interval under lambda; Multivariate Cox analysis of DE-TMRGs. **(B)** K-M curves for different risk groups in TCGA-LSCC samples. **(C)** ROC curves for different risk groups in TCGA-LSCC samples. **(D)** Risk score between Disease Free and Recurred/Progressed groups in TCGA-LSCC samples. **(E)** Disease Free and Recurred/Progressed percentage between different risk groups in TCGA-LSCC samples. **(F)** ROC curves, K-M curves, Risk score and percentage between different risk groups were validated in GSE27020 dataset. TMRGs, tryptophan metabolism-related genes; LSCC, laryngeal squamous cell carcinoma; ROC, Receiver operating characteristic; K-M, Kaplan-Meier.

Based on 98 DE-TMRGs, 14 genes were discovered using univariate Cox regression (HR ≠ 1, P < 0.05) in TCGA-LSCC. The LASSO method further refined this list to 12 genes based on a minimum lambda of 0.02907679. Subsequently, five prognostic genes (*SERPINA1*, *TMC8*, *RENBP*, *SDS*, and *FAM107A*) were determined through multivariate Cox regression analysis (HR ≠ 1, P < 0.05) (Figure 3A). Besides, the high-risk subgroup exhibited a substantial lower survival (Figure 3B). The area under the curve (AUC) values for 1- (0.88), 3- (0.82) and 5- (0.76) year all surpassed 0.7, indicating that the risk model performed exceptionally well in predicting LSCC patients. (Figure 3C). Undoubtedly, risk score differed sensibly between Disease Free and Recurred/Progressed groups (Figure3D), and the percentage of Recurred/Progressed patients with high risk was higher than that in Disease Free (Figure 3E). The results were further validated in GSE27020 (Figure 3F).

### 3.4 The risk score was defineded as independent prognosis factor, and the risk model was involved in metabolism related pathways

**FIGURE 4:**
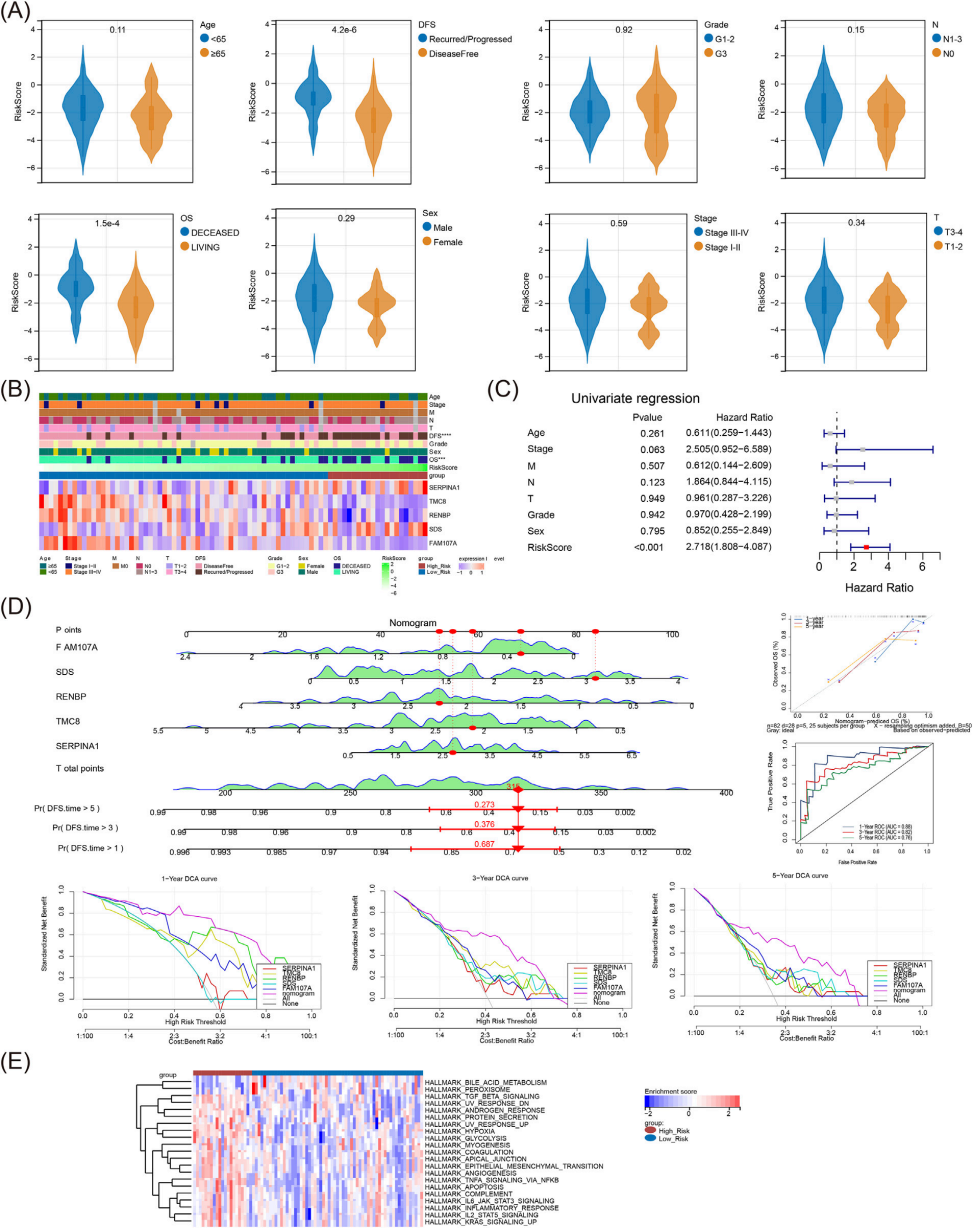
The risk score was defined as an independent prognosis factor, and the risk model was involved in metabolism related pathways: **(A)** Various clinicopathological groups derived from TCGA-LSCC cohort are compared pairwise in the parameter of risk score. **(B)** Heat map of risk model genes expressions in different risk groups and different clinical characteristics. **(C)** Univariate Cox analysis of independent prognostic factors. **(D)** Nomogram model; The nomogram showed the most powerful capacity for survival prediction; ROC curves of the nomogram; DCA curves of the nomogram. **(E)** The different Hallmark pathways in high-risk and low-risk groups displayed as a heatmap. LSCC, laryngeal squamous cell carcinoma; DCA, Decision curve analysis; ROC, Receiver operating characteristic.

Violin plot showed notable difference in risk score both between OS and DFS subgroups (Figure 4A), and heat map illustrated the prognostic gene expressions in different risk subgroups and different clinical features (Figure 4B). The independent prognosis factor risk score, was finally obtained through univariate Cox (Figure 4C). Subsequently, the nomogram indicated that the risk model had a good capacity to predict patients with LSCC, and the results were further supported by the calibration, ROC, and decision curves (Figure 4D). Moreover, the risk model was engaged in xenobiotic metabolism, fatty acid metabolism, bile acid metabolism, heme metabolism, IL6 JAK stat3 signaling and other hallmark pathways (Figure 4E).

### 3.5 The risk model had a important effect in the immune microenvironment and chemotherapy of LSCC

**FIGURE 5:**
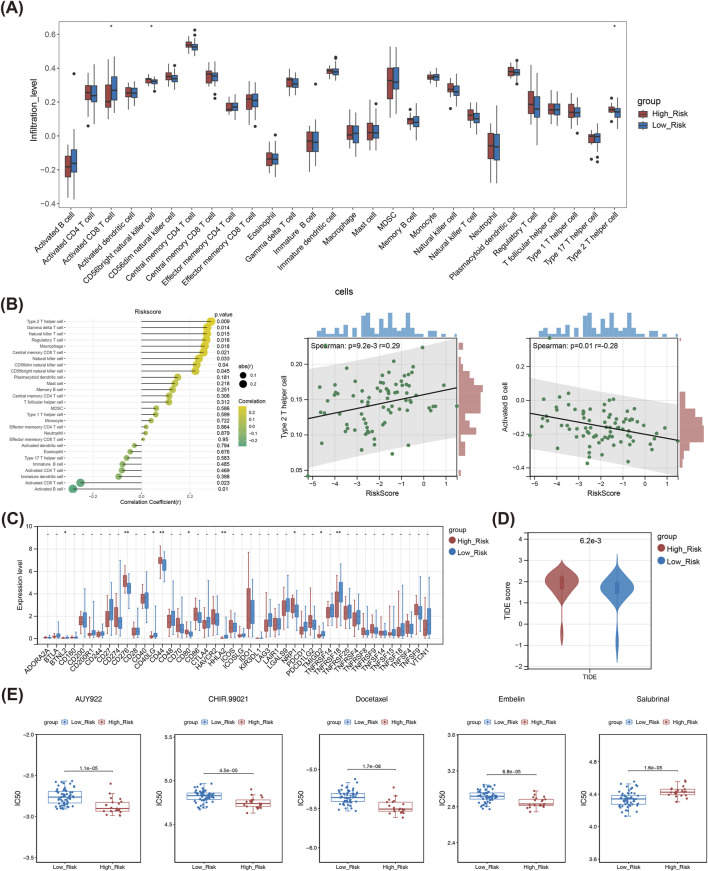
Immune microenvironment analysis of the TCGA-LSCC cohort: **(A)** Box plot of infiltration abundance of 28 immune cells in high and low-risk groups. **(B)** Correlation analysis between 28 immune cell and risk score; Correlation scatter plot between risk score and Type 2 helper T cell; Correlation scatter plot between risk score and activated B cell; **(C)** Differentially expressed immune checkpoint genes in high and low-risk groups. **(D)** The TIDE score in high and low-risk groups. **(E)** The estimated IC_50_ values for top five drugs of significant differences between high and low-risk groups. * means p < 0.05,** means p < 0.01,*** means p < 0.001,**** means p < 0.0001. LSCC, laryngeal squamous cell carcinoma; IC_50_, half maximal inhibitory concentration; TIDE, tumor immune dysfunction and exclusion.

In box plot, it could be seen that the infiltration levels of CD56 bright natural killer (NK) cell, Type 2 helper T cell, and Activated CD8 T cell differed notably between two risk subgroups (Figure 5A). Besides, the risk score showed a noteworthy positive correlation with Type 2 helper T cell, and had a notable adverse correlation with Activated B cell (Figure 5B). A total of nine immune checkpoints exhibited sensible expression disparities among high/low-risk subgroups, including BTNL2, CD276, CD44, HHLA2, and TNFRSF18, *etc.* (Figure 5C). Obviously, the TIDE score was significantly lower in the low-risk subgroup compared to the high-risk group, indicating a lower likelihood of immune evasion and better response to immunotherapy among low-risk LSCC patients (Figure 5D). Eventually, the study found that patients with low-risk were notably sensitive to Salubrinal, Lenalidomide, Metformin and others, while those with high-risk were noticeably sensitive to Docetaxel, AUY922, Embelin, *etc.* (Figure 5E).

### 3.6 Immunological microlandscape and drug sensitivity between clusters

**FIGURE 6:**
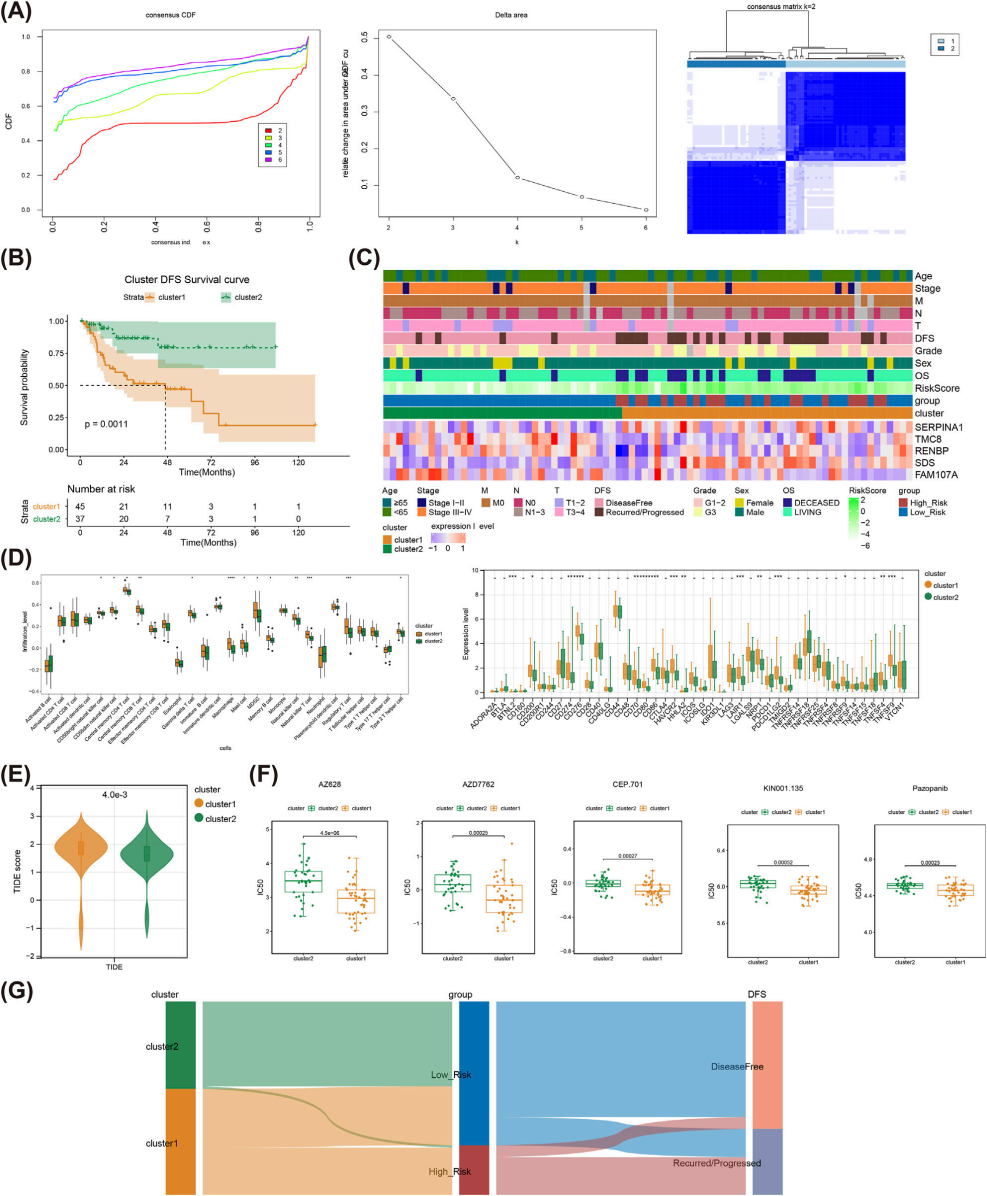
Consensus clustering analysis of the TCGA-LSCC cohort. **(A)** Cumulative distribution function curves for samples that are from TCGA-LSCC cohort; Curves for the delta area under the cumulative distribution function curves for samples that are from TCGA-LSCC cohort; The two sample clusters (*k* = 2) was displayed as a heat map. **(B)** The prognosis of two TCGA-LSCC clusters is displayed as a K-M curve. **(C)** The risk model genes expressions in different clusters and clinical characteristics as a heat map. **(D)** Differentially infiltrated immune cells between different clusters; Differentially expressed immune checkpoints between different clusters. **(E)** The TIDE score between different clusters. **(F)** The estimated IC_50_ values for top five drugs of significant differences between different clusters. **(G)** The relationship among molecular clusters, high/low-risk groups, and DFS status are displayed as sankey diagram. LSCC, laryngeal squamous cell carcinoma; IC_50_, half maximal inhibitory concentration, TIDE, tumor immune dysfunction and exclusion; K-M, Kaplan-Meier.

Through consensus clustering analysis, the LSCC samples were classified into two clusters (Figure 6A), and cluster1 had a poorer survival state than that of cluster 2 (Figure 6B). The heat map illustrated the prognostic gene expressions in different clusters and clinical characteristics (Figure 6C). Moreover, the infiltration levels of 13 immune cells like Macrophage and Regulatory T cell, and expression levels of 15 immune checkpoints including BTNL2, TNFSF9 and others were sensibly different between cluster1 and cluster2 (Figure 6D). Meanwhile, the TIDE score of cluster 1 was notably higher than that of the cluster 2, suggesting a higher probability of LSCC immune evasion of cluster 1 patients (Figure 6E). Apparently, the patients in cluster 2 were sensibly sensitive to Rapamycin, Salubrinal, Tipifarnib and so on, while patients in cluster 1 were more responsive to AZ628, Pazopanib, AZD7762, *etc.* (Supplementary Figure S1, Figure 6F). Sankey diagram demonstrated the relationship among molecular clusters, high/low-risk subgroups, and DFS status (Figure 6G).

### 3.7 Expression validation

**FIGURE 7:**
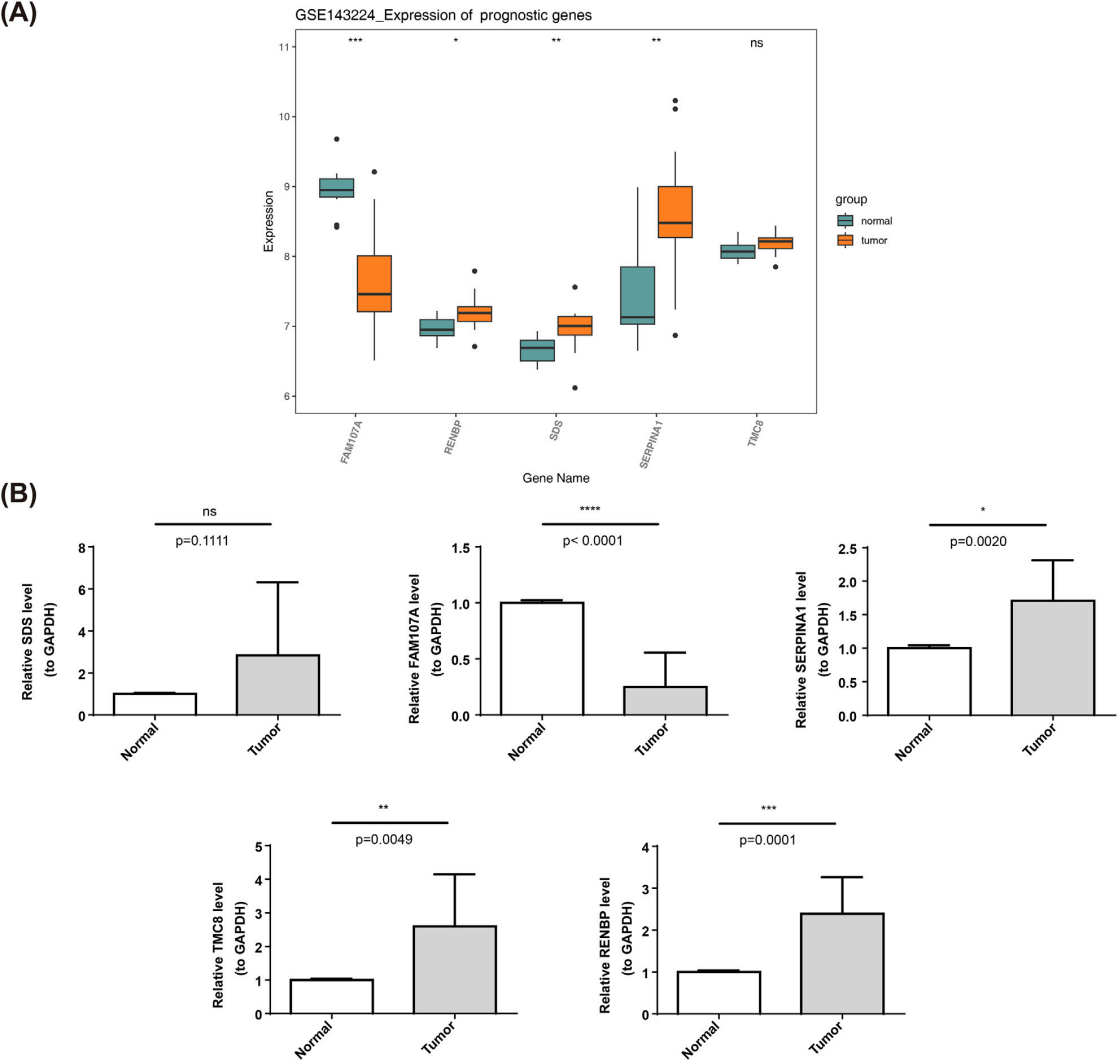
**(A)**: The expression of prognostic gene in GSE143224. **(B)**: Expression validation of prognostic gene in our study.

To investigate the expression of prognostic gene (*SERPINA1*, *TMC8*, *RENBP*, *SDS*, and *FAM107A*), firstly, the three prognostic genes (*RENBP*, *SDS*, and *SERPINA1*) were remarkably upregulated in the tumor group compared with the normal in GSE143224 (P < 0.05), in contrast, *FAM107A* exhibited significantly higher expression levels in normal samples (P < 0.05). The expression level of *TMC8* was higher in the tumor group samples than in the normal group, but the difference was not significant (Figure 7A). Meanwhile,we examined their expression in LSCC tissue by qRT-PCR and compared it with normal tissue samples, we obtained similar conclusions. Specifically, our data showed a significant decrease in the expression of *FAM107A* and a increase in the expression of *SERPINA1*, *TMC8*, *RENBP*, in LSCC patients compared to normal (Figure 7B).

## 4 Discussion

LSCC is the most common pathological type of LCa and also one of the most common malignant tumors of the head and neck (Johnson et al., 2020; Cohen et al., 2019). The incidence of LSCC has been on the rise in recent years, both in China and other countries, and its poor prognosis and high mortality rate have become a major risk factor for human health (Echanique et al., 2021; Costa et al., 2021; Deng et al., 2020). Despite the continuous improvement in the diagnosis and treatment of LSCC, including surgery, radiotherapy, chemotherapy, neoadjuvant therapy, immunotherapy, or strategies combining different treatment modalities,the overall survival rate has not been significantly improved (Qin et al., 2021; Bryan et al., 2016; Karam and Raben, 2019; Guidi et al., 2018). Due to the insidious onset of LSCC, which has already progressed to mid and late stages in most patients at the time of diagnosis, and the high recurrence rate, the 3, 5-year DFS of LSCC has not been significantly improved either (Volkov, 1972; Obid et al., 2019). Therefore, searching for new targets, early prediction, early diagnosis, early intervention, and providing new diagnostic and therapeutic approaches for LSCC may be important means to improve the prognosis of LSCC patients (Sung et al., 2021).

Trp metabolism produces a variety of active substances with physiological effects, which are important for maintaining cell activation and proliferation (Neavin et al., 2018; Conejos et al., 2021). Trp and its metabolites have diverse biological effects in the regulation of multiple diseases (Conejos et al., 2021; Ding et al., 2020; Wu et al., 2020). In particular, Trp and its metabolites play an important role in tumor formation and evolution (Kwiatkowska et al., 2021; Tanaka et al., 2021). Perez L et al. (Perez-Castro et al., 2023) showed that Trp metabolites activate the aromatic receptor (AHR), increase malignant degree, and inhibit anti-tumor immune responses. In a study of oral squamous cell carcinoma (OSCC), Tryptophanyl-tRNA synthetase (*TrpRS*) expression was upregulated in tumor tissues, and the expression level of TrpRS correlated with the clinicopathological features of OSCC patients (Lee et al., 2015). Another study regarding head and neck squamous carcinoma (*HNSC*) showed that *IDO* and *TDO2* are indicators of immunosuppression and poor survival in HNSC patients, and that *IDO1* in *HNSC* is negatively correlated with programmed cell death protein ligand 1 (*PDL-1*) (Riess et al., 2020). Additionally, studies revealed that TMRGs also play significant roles in LSCC and OSCC. For instance, drugs that inhibit the overexpression of Akt1 (RAC-α serine/threonine-protein kinase) and Akt2 (RAC-β serine/threonine-protein kinase) without reducing MAOB expression could serve as lead compounds for the treatment of oral squamous cell carcinoma (OSCC) (Sharif Siam et al., 2021). Furthermore, ACAT1 inhibitors have been shown to enhance the therapeutic efficacy of CSCs-DC vaccines in a mouse model of postoperative recurrence of head and neck squamous cell carcinoma (HNSCC), suggesting that ACAT1 may play an important role in cancer immunotherapy (Chen et al., 2017). Additionally, both low and high expression levels of ALDH2 have been associated with tumor progression, depending on the type of cancer, indicating that ALDH2 is a potential therapeutic target for cancer treatment (Zhang and Fu, 2021).

**FIGURE 8:**
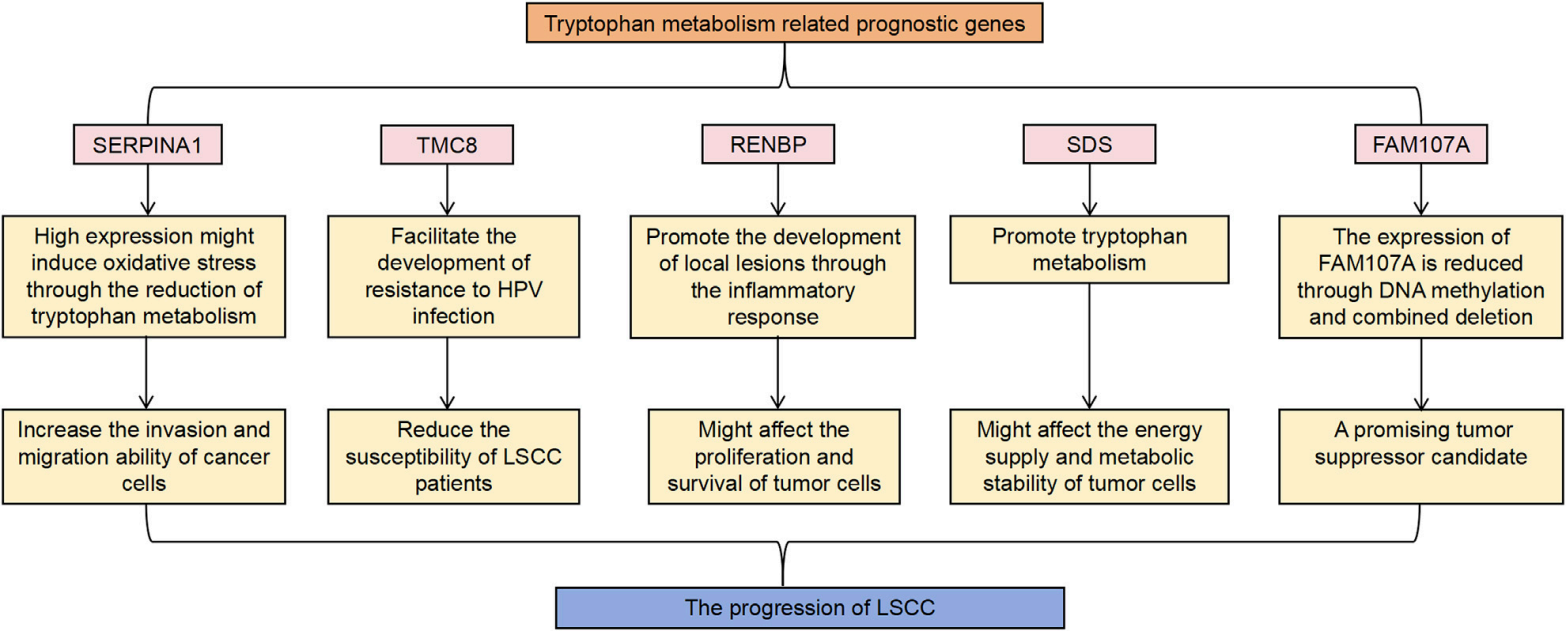
The potential pathway interactions of TMRGs in LSCC.

In this study, we analyzed the RNA-seq data, copy number, somatic mutation data and clinicopathological features of the TCGA-LSCC dataset to explore the mechanisms related to Trp metabolism in LSCC, and finally identified five genes (*SERPINA1*, *TMC8*, *RENBP*, *SDS*, and *FAM107A*) to establish a risk model. Tryptophan metabolism, particularly through the kynurenine pathway, plays a crucial role in shaping the tumor immune microenvironment (Qin et al., 2021). Key enzymes such as IDO1 and TDO2 catalyze the conversion of tryptophan to kynurenine, producing immunosuppressive metabolites that inhibit anti-tumor immune responses (Prendergast et al., 2014). In LSCC, the upregulation of these enzymes may lead to increased immune evasion and tumor progression. Additionally, TMRGs may affect the infiltration of immune cells such as Tregs and MDSCs, which contribute to an immunosuppressive microenvironment (Perez-Castro et al., 2023). The potential pathway interactions of TMRGs in LSCC are illustrated in Figure 8. In addition, two molecular clusters related to Trp metabolism were also identified.

The *SERPINA1* encodes the alpha-1 antitrypsin (AAT) protein, which is involved in the regulation of enzyme activity and protein metabolism, as well as cell growth, differentiation, motility and signaling (Ando et al., 2021; Lim et al., 2020; Cipriani et al., 2018). Previous research findings have indicated that there is a significant upregulation of *SERPINA1* in various types of tumor tissues, which has been strongly linked to the advancement and prognosis of tumors. Jiang and Hu (2020) found that *SERPINA1* expression was linked to the development of gastric cancer and enhanced the invasion and migration of the disease’s cells. As a result, it may offer a viable target for the disease’s prevention. Kwon et al. (2015) found that *SERPINA1* expression was linked to poor prognosis of colorectal cancer, as well as tumor stage, and lymph node metastasis, and that overexpression of *SERPINA1* increased the capacity of cancer cells to invade and migrate, whereas silencing of *SERPINA1* expression decreased this capacity. In hepatocellular carcinoma, Dongre and Weinberg (2019) reached a similar conclusion that patients with high expression of *SERPINA1* in hepatocellular carcinoma tissues may have a higher risk of recurrence and metastasis. The expression level of *SERPINA1* in oral tumor tissues was notably higher than normal tissues, which was associated with the progression, metastasis and prognosis of the tumor (Kawahara et al., 2016). In our study, we used qRT-PCR to detect the expression level of *SERPINA1* in tumor and adjacent tissues of LSCC patients, and the results showed that the expression level of *SERPINA1* in LSCC was significantly higher, which was in line with the results of other scholars’ studies.

The mammalian transmembrane channel-like (TMC) gene family consists of eight genes, ranging from TMC 1 to 8, and *TMC8* is also known as *EVER2*. It is widely acknowledged that individuals diagnosed with HNSC frequently exhibit HPV infection, and studies in recent years have suggested that common variants of *TMC8* are noteworthily related to HPV infection and HNSC patients prognosis (Lin et al., 2021). Liang et al. (2015) also have reported a correlation between genetic variation in *TMC8* and the occurrence of high-risk HPV infection as well as its role in the development of HNSC, and it is also a fundamental regulatory factor for the persistence and carcinogenicity of HPV infection. In addition, *TMC8* has the potential to impact tumor behavior by modulating immune pathways; The presence of *TMC8* in tumor tissues showed a significant and positive correlation with the infiltration of various immune cells, including B cells, CD4^+^ T cells, macrophages, CD8^+^ T cells, and dendritic cells, and *TMC8* may increase the infiltration of cytotoxic T cells and B lymphocytes by influencing the functionality of CD4^+^ T cells (Liang et al., 2015). According to a study by Lin et al. (2021) *TMC8* expression was shown to be considerably higher in HPV-positive HNSC patients. This finding indicated a positive link between Th2 infiltration and B cell enrichment, indicating that *TMC8* may operate as a barrier to HPV through Th2 cells. We propose that *TMC8* could potentially contribute to the development of resistance against HPV infection, consequently reducing the vulnerability of LSCC patients.

*RENBP* encodes renin-binding protein, an important regulator in the renin-angiotensin-aldosterone system, which is responsible for participating in blood pressure regulation and maintenance of sodium homeostasis. *RENBP* has been proved to be expressed in mouse lungs and may promote local lesion development through an inflammatory response (Huffnagle et al., 2017), so we hypothesized that micro-metabolic changes induced by inflammation in LSCC may affect *RENBP* gene expression and consequently affects tumorigenesis.

The family with sequence similarity 107 member A (FAM107A) gene, also known as DRR1 or TU3A, a specific set of codons encodes multiple amino acids. Previously published studies have shown that *FAM107A* has reduced expression in various tumor tissues, including prostate cancer, hepatocellular carcinoma, lymphoma and non-small cell lung cancer, which exhibits the properties of an oncogene (Vanaja et al., 2006; Udali et al., 2015; Lawrie et al., 2018; Pastuszak-Lewandoska et al., 2015); while *FAM107A* was highly expressed in the aggressive component of gliomas and was associated with increased aggressiveness and disease progression in neuroblastoma, meningioma and malignant glioma (Mu et al., 2017; Asano et al., 2010; Fevre-Montange et al., 2009; Nakajima and Koizumi, 2014). The study of Kiwerska et al. (2017) found that *FAM107A* functioned as an oncogene in LSCC, and it led to the decrease of its expression in LSCC through DNA methylation and combined deletion. In the present study, the expression level of *FAM107A* in tumor tissues was significantly lower than that in the adjacent normal tissues, which was in line with the findings of other scholars.

Compared with other genes, *SDS* has not been deeply studied in LSCC, and the specific mechanism of *SDS* as a pro-carcinogenic gene in LSCC still needs to be further explored in the future.

We further confirmed that risk score alone was an independent prognostic factor by univariate Cox analysis of clinical characteristics and risk score, and in turn created a nomogram for prediction of risk and survival probability in LSCC. Hallmark pathway enrichment analysis reveals the risk model was also engaged in bile acid metabolism, fatty acid metabolism, xenobiotic metabolism, heme metabolism, IL6 JAK stat3 signaling and other hallmark pathways.

Immunotherapy is currently a more promising means of tumor treatment, especially immune checkpoint-based immunotherapy, which has made significant breakthroughs in the treatment of various tumors and reshaped the landscape of tumor treatment (Marabelle et al., 2020; Kruger et al., 2019; Ribas and Wolchok, 2018). In this study, we found that a total of nine immune checkpoints were sensitively expressed in high/low-risk subgroups. The high risk subgroup had much higher levels of immune checkpoint gene expression, such as Neuropilin-1 (*NRP1*). NRP1 is a neuronal and endothelial cell receptor essential for normal embryonic development and angiogenesis, whereas it suppresses anti-tumor immune response in the formation and development of malignant tumors, which correlates with poor clinical outcome (Delgoffe et al., 2013; Overacre-Delgoffe et al., 2017). On the one hand, *NRP1* promotes tumor cell growth through multiple pathways, such as neoangiogenesis, metastasis; on the other hand, *NRP1* facilitates immune escape by regulating intra-tumor Treg cells and CD8^+^ T and orchestrating multiple inhibitory processes in the tumor microenvironment (TME) (Overacre-Delgoffe et al., 2017; Liu et al., 2020). As a member of the B7/CD28 family, the molecular structure of human endogenous retrovirus-H long terminal repeat-associated protein 2 (*HHLA2*) shares some homology and similarity with other B7 family members, such as PD-L1, and exhibits both co-stimulatory and co-inhibitory effects on T cells (Janakiram et al., 2017; Xiao and Freeman, 2015) *TMIGD2* is one of the specific receptors for *HHLA2*, often also known as *CD28H* (Zhu et al., 2013). It has been discovered that several solid tumors have high expressions of HHLA2, including breast cancer (Liu et al., 2017), osteosarcoma (Koirala et al., 2016), colon cancer (Zhu and Dong, 2018), clear cell carcinoma of the kidney (Chen et al., 2019), and bladder cancer (Lin et al., 2019), and so on. Based on this, *HHLA2/TMIGD2* is expected to be one of the new pathways and targets for tumor immunotherapy, especially providing new ideas for PD-L1-negative tumor immunotherapy. Some published research indicated that *HHLA2* may have different effects on prognosis in different types of tumors. On the one hand, overexpression of HHLA2 in pancreatic, ampullary and ovarian carcinoma is closely associated with a favorable prognosis (Boor et al., 2020; Xu G. et al., 2021). On the other hand, co-expression of *HHLA2* and *PD-L1* in renal clear cell carcinoma shows a poor prognosis (Zhou et al., 2020). In our study, *HHLA2* was highly expressed in patients with low-risk, suggesting that LSCC patients with low-risk may benefit from immunotherapy based on this target. *CD40LG* (also known as *CD154*) is the ligand gene for *CD40*, belonging to the tumor necrosis factor (TNF) gene superfamily, predominantly expressed on activated T cells and platelets (Kuhn et al., 2019). It is also involved in tumor pathogenesis and anti-tumor immunity (Hassan et al., 2015). *CD40* ^*−*^ *CD40LG* interaction is an important signal for B-cell proliferation, activation biomarker expression and immunoglobulin production, which directly inhibits the growth of *CD40*-positive tumor cells and may indirectly inhibit tumor growth by downregulating the immune response (Laman et al., 2017; Jansen et al., 2016). Yuan et al. (2021) found that *CD40LG* is a valuable prognostic molecular marker in breast cancer, and its low expression is associated with poor outcomes. In our study, we found that CD40LG was lowly expressed in the high risk subgroup, which may be associated with a poorer prognosis in these patients. For the TIDE score, it was higher in patients with high-risk than low-risk, suggesting that high-risk patients may have a higher likelihood of immune escape in response to immune checkpoint therapy, which may have a negative impact on the effect of immunotherapy in these patients.

To explore the role of risk model in LSCC chemotherapy and find chemotherapy-sensitive drugs in high/low-risk subgroup, we performed a drug sensitivity analysis and found that patients in the low-risk subgroup were sensitive to Salubrinal, while those in the high-risk subgroup were sensitive to these drugs including Docetaxel, AUY922, Embelin, and CHIR.99021. Salubrinal is a selective inhibitor of eIF2α that acts by inducing phosphorylation and inhibiting dephosphorylation of eIF2α. Salubrinal was found to act differently when combined with different types of chemotherapeutic agents, it can enhance the inhibitory effect of drugs such as doxorubicin on tumors (Jeon et al., 2016); whereas in combination with platinum-based drugs, Salubrinal attenuates the antineoplastic efficacy of platinum-based chemotherapeutic agents (Chen et al., 2011; Feng et al., 2013). Therefore, when selecting chemotherapeutic drugs for patients in the high-risk subgroup, attention should be paid to the rational selection of drugs to maximize the antitumor activity of different types of drugs.

Based on consensus clustering analysis, we identified two different clusters, and the cluster 1 had worse DFS compared to the cluster 2. The proportions of immune cells such as MDSC, Macrophage, and Regulatory T cells were also higher in cluster 1 than in cluster 2 subtype, which is important for the formation of an immunosuppressive tumor microenvironment and the inhibition of migration and infiltration of immune cells into tumor tissues, and this may be one of the reasons for the worse DFS of the cluster 1, which is in line with the studies of other scholars (Sica and Massarotti, 2017; Wang et al., 2021).

Trp metabolism is closely associated with the formation of the tumor immune microenvironment and impacts anti-tumor immune responses through multiple pathways. Increased activity of rate-limiting enzymes in Trp metabolism leads to Trp depletion in the tumor microenvironment, which in turn induces cell-cycle arrest of peripheral T cells and promotes the generation of Tregs (Wolf et al., 2015; Fallarino et al., 2006). Moreover, Kyn, a metabolite of Trp metabolism, can activate AHR on CD4^+^ T cells and induce differentiation of CD4^+^ T cells to regulatory T cells (Mezrich et al., 2010). Within the tumor microenvironment, Tregs inhibit the function of effector T cells by secreting inhibitory cytokines or interacting with antigen-presenting cells, thereby modulating anti-tumor immune responses. This aligns with the immune cell infiltration pattern we observed in cluster 1, suggesting that Trp metabolism plays a pivotal role in shaping an immunosuppressive microenvironment. We further examined the expression of immune checkpoint genes across various clusters. Intriguingly, a higher degree of suppression was observed in cluster 2 compared to cluster 1, suggesting that a favorable clinical outcome for cluster 2 may be achieved through immune checkpoint blockade rather than immune cell infiltration, and this is similar to the study by Luo P et al. on Trp metabolism in gastric cancer (Luo et al., 2023). Meanwhile, the TIDE score of cluster 1 was significantly higher than that of cluster 2, indicating that patients in cluster 1 are more likely to be refractory to immunotherapy, leading to a poorer prognosis. In the analysis of chemotherapeutic drug sensitivity between the two molecular subtypes, we found that patients in cluster 1 were more sensitive to drugs such as AZ628, AZD7762, CEP.701, KIN001.135, and Pazopanib, while patients in cluster 2 were more sensitive to rapamycin, Salubrinal, and Tipifarnib.

Our study suggests that high-risk LSCC patients may have a poorer response to immunotherapy, as indicated by higher TIDE scores and lower immune cell infiltration. While our findings are based on bioinformatics analysis and require experimental validation, they provide a potential framework for identifying patients who may benefit less from standard immunotherapy approaches. In clinical settings, this could help in stratifying patients for alternative or combination therapies. For instance, for high-risk patients, personalized immunotherapy strategies could be developed. In addition to conventional immunotherapy, combination treatments (such as chemotherapy, targeted therapy, or other immunotherapeutic approaches) could be considered to improve treatment outcomes. Future research should focus on validating these findings through prospective clinical trials and functional studies to explore the therapeutic potential of targeting specific TMRGs or immune checkpoints in LSCC.

Nevertheless, it is important to acknowledge certain limitations in our investigation. Firstly, the data utilized in our study were retrospectively collected from the TCGA and GEO databases. Therefore, the potential bias in case selection might affect the results. Secondly, these data were analyzed only by bioinformatics, and future investigations should include relevant *in vivo* and *in vitro* validation experiments to confirm the efficacy of risk modeling. In addition, the molecular mechanisms associated with Trp metabolism in LSCC remain to be further validated. In addition, the molecular mechanisms associated with Trp metabolism in LSCC remain to be further validated. Moreover, the sample size of our qRT-PCR validation was relatively small, which limits the robustness of our findings. Future studies should validate the identified TMRGs in larger patient cohorts. Furthermore, functional assays, such as gene knockdown or overexpression studies, are needed to establish the mechanistic roles of these genes in LSCC progression. Additionally, immunohistochemistry or Western blot analysis should be incorporated to verify protein-level expression and localization.

## 5 Conclusion

In this study, five TMRGs (*SERPINA1*, *TMC8*, *RENBP*, *SDS* and *FAM107A*) were identified to further establish a risk model, which had specifically good predictive performance, and can provide an effective method for evaluating the prognosis LSCC patients. The nomogram based on the five TMRGs can provide a concise and digital predictions to patients through simple calculations. Our study also reveals the clinical significance of Trp metabolism and its underlying association with LSCC, which may provide a new approach for personalized comprehensive treatment of LSCC patients.

## Data Availability

The datasets presented in this study can be found in online repositories. The names of the repository/repositories and accession number(s) can be found in the article/Supplementary Material.

## Abbreviations

PCOS, Polycystic ovary syndrome; DEGs, Differentially expressed genes; HA, Hyperandrogenism; SVM-RFE, Support vector machine recursive feature elimination; DCA, Decision curve analysis; FC, Fold change; IR, Insulin resistance; LASSO, Least absolute shrinkage and selection operator; MSigDBq, Molecular signatures database; qRT-PCR, Quantitative real-time polymerase chain reaction; PCA, Principal component analysis; GEO, Gene expression omnibus; GO, Gene ontology; KEGG, Kyoto encyclopedia of genes and genomes; AUC, Area under curve; BP, Biological process; MF, Molecular function; CC, Cellular component; ROC, Receiver operating characteristic; ssGSEA, Single-sample gene set enrichment analysis; TFs, Transcription factors; CTD, Comparative toxicogenomics database; CRG, Cell adhesion-related genes.
